# Supplementary figures and images for: Feedback Control of a Two-Component Signaling System by an Fe-S-Binding Receiver Domain
Source: mBio. 2020 Mar 17;11(2):e03383-19. doi: 10.1128/mBio.03383-19 (PMC7078487; doi:10.1128/mBio.03383-19)

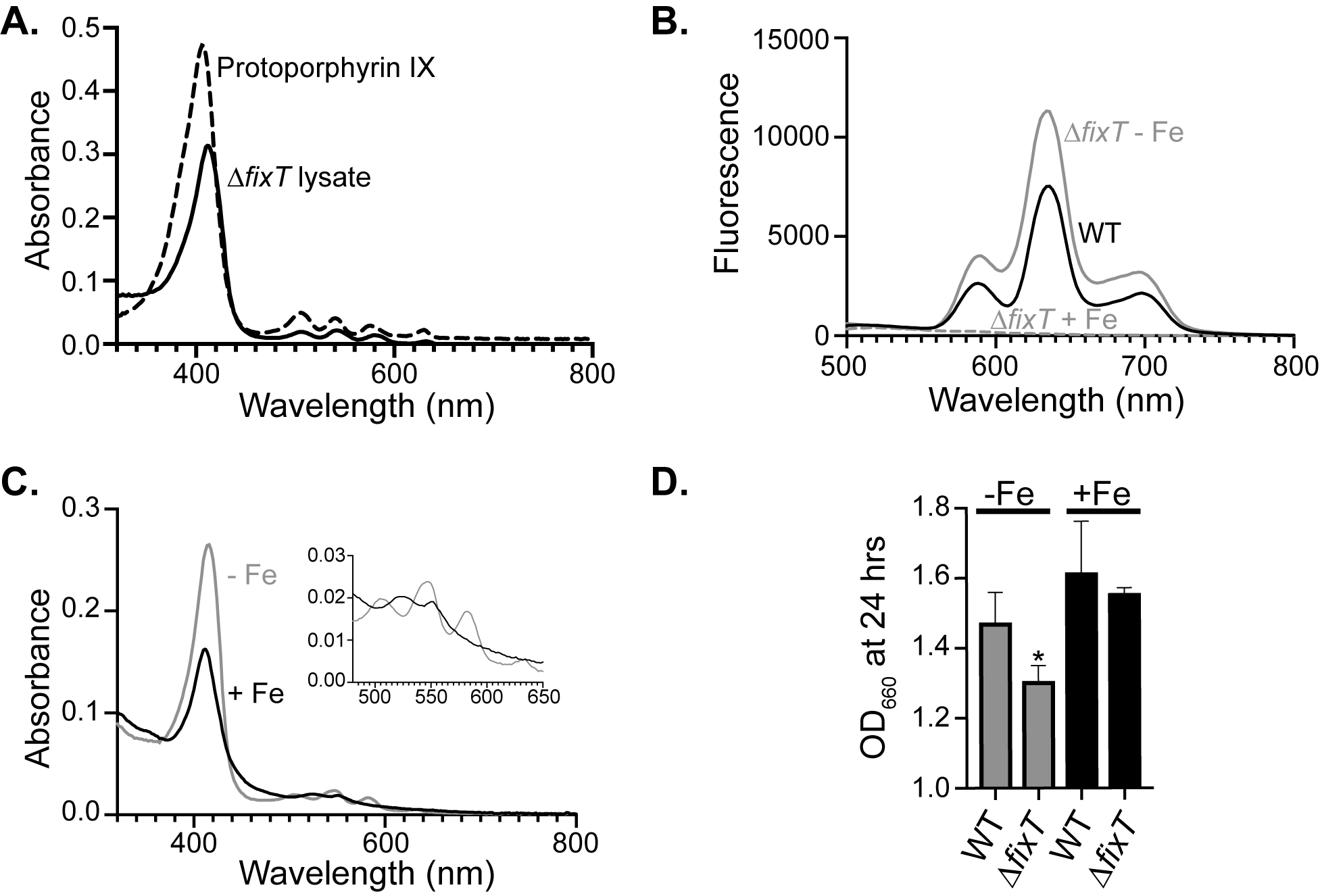

Supplement: FIG S1 [file mBio.03383-19-sf001.tif]

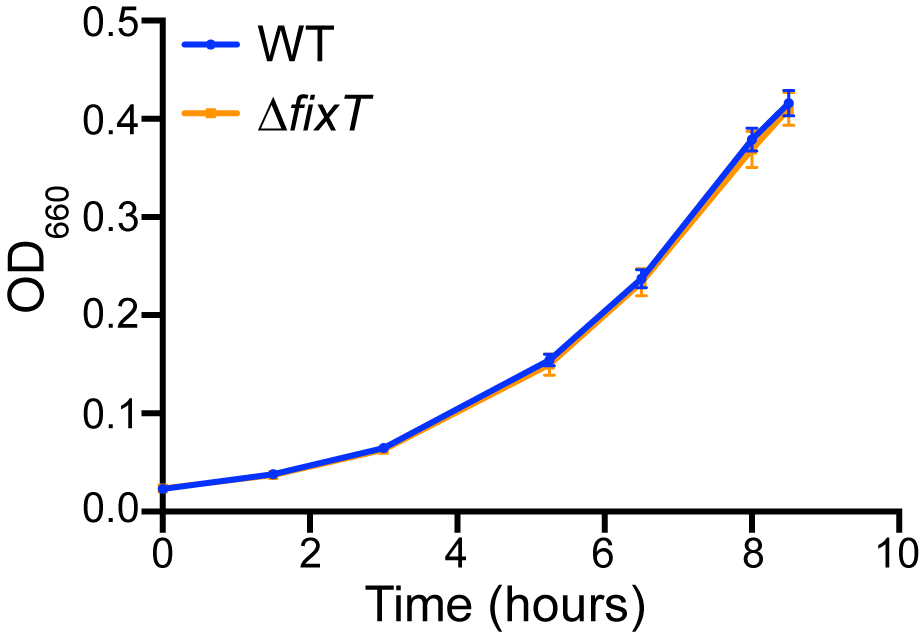

Supplement: FIG S2 [file mBio.03383-19-sf002.tif]
