# Supplementary material for: Feedback Control of a Two-Component Signaling System by an Fe-S-Binding Receiver Domain
Source: mBio. 2020 Mar 17;11(2):e03383-19. doi: 10.1128/mBio.03383-19 (PMC7078487; doi:10.1128/mBio.03383-19)
Supplement: TABLE S1 [file mBio.03383-19-st001.docx]

**Table S1: Strains and plasmids used in this work**

| ***E. coli* strains with plasmids** | | | | |
| --- | --- | --- | --- | --- |
| **Strain #** | **Genotype** | **Comments** | | **Source** |
| FC929 | TOP10 | Cloning strain | | Invitrogen |
| FC3 | MT607 / pRK600 | Helper strain for tri-parental matings | | (1) |
| FC803 | Rosetta (DE3) pLysS | Strain for protein expression from T7 promoter | | Novagen |
| ***Caulobacter e*xpression plasmids** | | | | |
| MTLS4423 | TOP10 / pMT805 (pBXMCS-2) | Replicating plasmid (mid copy, BBR origin) with xylose inducible promoter, Chlor^R^ | | (2) |
| FC3396 | TOP10 / pMT805-*fixT* | Cloning Primers:   \| 5’-gttgcatATGCGACGCATGTGCGAC-3’  5’-caacgagctcctaGAGCCCCGGCG-3’ \| \| --- \| | | This work |
| FC3397 | TOP10 / pMT805-*fixT(C53S)* | Generated from FC3396 | | This work |
| FC3398 | TOP10 / pMT805-*fixT(C56S)* | Generated from FC3396 | | This work |
| FC3399 | TOP10 / pMT805-*fixT(C59S)* | Generated from FC3396 | | This work |
| FC3400 | TOP10 / pMT805-*fixT(C64S)* | Generated from FC3396 | | This work |
| FC3401 | TOP10 / pMT805-*fixT(4C)* | Mutant containing all mutations in above 4 plasmids, Generated from FC3396 | | This work |
| **Allele Replacement Plasmids** | | | | |
| FC55 | DH10B / pNPTS138 | Allele replacement plasmid, Kan^R^, SacB | | M.R.K. Alley |
| FC106 | TOP10/ pNPTS138-*∆fixT* |  | | (3) |
| FC3402 | TOP10 / pNPTS138-*∆hslU* | knockout allele contains first 6 and last 3 codons of *hslU*. 5’ end (5’-ATGACCGAG…), 3’ end (…ATTTTGtag-3’) | | This work |
| FC3403 | TOP10 / pNPTS138-*∆ftsH* | knockout allele contains first 4 and last 107 codons of *ftsH*. 5’ end (5’-ATGAATTTC…), 3’ end (…ACCGCCtga-3’) | | This work |
| FC2256 | TOP10 / pNPTS138-∆*clpA* | knockout allele contains first 13 and last 11 codons of *clpA*. 5’ end (5’-TTGCCCTCT…), 3’ end (…GCCGAAtag-3’) | | This work |
| **Transcriptional *lacZ* fusion plasmids** | | | | |
| FC94 | TOP10 / pRKlac290-P*fixK* | Plasmid containing P*_fixK_-lacZ* transcriptional fusion, tet^r^ | | (3) |
| **Heterologous protein expression plasmids** | | | | |
| FC3404 | DH5α / pET23b-H_6_-*smt3* | Plasmid to express proteins fused to the H_6_-SUMO tag | | (4) |
| FC3249 | DH5α / pET23b-H_6_-*smt3* V2 | Derived from FC3404, contains additional restriction sites for cloning | | Breann Brown |
| FC3405 | DH5α / pET23b-Ulp1 | Plasmid to express H_6_-Ulp1 for cleavage of SUMO tag | | (4) |
| FC3406 | TOP10 / pET23b-H_6_-*smt3-fixT* | *fixT* insert amplified from FC3396 | | This work |
| FC3407 | TOP10 / pET23b-H_6_-*smt3-fixT(C64S)* | *fixT* insert amplified from FC3400 | | This work |
| FC3408 | TOP10 / pET23b-H_6_-*smt3-fixT(4C)* | *fixT* insert amplified from FC3401 | | This work |
| FC3409 | TOP10 / pET23b-H_6_-*smt3-fixL_118-495_* | Cloning Primers:  5’-gatcaccggtggtGCGGCGGCGGTCAATG-3’  5’-caatgagctctcaGTCATCGATGGTCTCC-3’ | | This work |
| FC3410 | TOP10 / pET23b-H_6_-*smt3-fixJ* | Cloning Primers:  5’-gatcaccggtggtATGACTGACGCCCC-3’  5’-gtgcggccgctcaGCCGCCGCGC-3’ | | This work |
| FC3411 | TOP10 / pET23b-H_6_-*smt3-fixJ(D55A)* | Generated from FC3410 | | This work |
| ***Caulobacter crescentus* strains** | | | | |
| **Strain #** | **Genotype** | | **Source** | **Figure** |
| FC19 | Wild type CB15 | | (5) | 1, 5, S1,S2 |
| FC3412 | CB15 / pMT805 | | This work | 1, 4 |
| FC98 | CB15 / pRKlac290-P*fixK* | | (3) | 1 |
| FC3413 | CB15 / pMT805 / pRKlac290-P*fixK* | | This work | 4 |
| FC13 | CB15 *∆fixT* | | (3) | 1, S1, S2 |
| FC3414 | CB15 *∆fixT /* pMT805 | | This work | 1, 5 |
| FC112 | CB15 *∆fixT /* pRKlac290-P*fixK* | | (3) | 1 |
| FC3415 | CB15 *∆fixT /* pMT805 */* pRKlac290-P*fixK* | | This work | 4 |
| FC3416 | CB15 *∆fixT* */* pMT805-*fixT* | | This work | 1, 5 |
| FC3417 | CB15 *∆fixT* */* pMT805-*fixT(4C)* | | This work | 5 |
| FC3418 | CB15 *∆fixT /* pMT805-*fixT /* pRKlac290-P*fixK* | | This work | 4 |
| FC3419 | CB15 *∆fixT /* pMT805-*fixT(C53S) /* pRKlac290-P*fixK* | | This work | 4 |
| FC3420 | CB15 *∆fixT /* pMT805-*fixT(C56S) /* pRKlac290-P*fixK* | | This work | 4 |
| FC3421 | CB15 *∆fixT /* pMT805-*fixT(C59S) /* pRKlac290-P*fixK* | | This work | 4 |
| FC3422 | CB15 *∆fixT /* pMT805-*fixT(C64S) /* pRKlac290-P*fixK* | | This work | 4 |
| FC3423 | CB15 *∆fixT /* pMT805-*fixT(4C) /* pRKlac290-P*fixK* | | This work | 4 |
| FC2264 | CB15 *∆lon* | | (6) | 5 |
| FC3424 | CB15 *∆fixT∆lon* | | This work | 5 |
| FC3425 | CB15 ∆*fixT∆lon /* pMT805-*fixT* | | This work | 5 |
| FC3426 | CB15 ∆*fixT∆lon /* pMT805-*fixT(4C)* | | This work | 5 |
| FC3427 | CB15 *∆fixT∆hslU* | | This work |  |
| FC3428 | CB15 *∆fixT∆hslU /* pMT805-*fixT* | | This work | 5 |
| FC2265 | CB15 *∆clpA* | | This work |  |
| FC3429 | CB15 *∆fixT∆clpA* | | This work |  |
| FC3430 | CB15 *∆fixT∆clpA* pMT805-*fixT* | | This work | 5 |
| FC2417 | CB15 *∆socAB∆clpX /* pAC152 | | (7) |  |
| FC3431 | CB15 *∆fixT∆socAB∆clpX /* pAC152 | | This work |  |
| FC3432 | CB15 *∆fixT∆socAB∆clpX /* pAC152 / pMT805-*fixT* | | This work | 5 |
| FC3433 | CB15 *∆fixT∆ftsH* | | This work |  |
| FC3434 | CB15 *∆fixT∆ftsH /* pMT805-*fixT* | | This work | 5 |

1. **Finan TM**, **Kunkel B**, **De Vos GF**, **Signer ER**. 1986. Second symbiotic megaplasmid in *Rhizobium meliloti* carrying exopolysaccharide and thiamine synthesis genes. J Bacteriol **167**:66–72.

2. **Thanbichler M**, **Iniesta AA**, **Shapiro L**. 2007. A comprehensive set of plasmids for vanillate- and xylose-inducible gene expression in *Caulobacter crescentus*. Nucleic Acids Res **35**:e137.

3. **Crosson S**, **McGrath PT**, **Stephens C**, **McAdams HH**, **Shapiro L**. 2005. Conserved modular design of an oxygen sensory/signaling network with species-specific output. Proc Natl Acad Sci U S A **102**:8018–8023.

4. **Wang KH**, **Sauer RT**, **Baker TA**. 2007. ClpS modulates but is not essential for bacterial N-end rule degradation. Genes Dev **21**:403–408.

5. **Poindexter JS**. 1964. Biologica properties and classification of the *Caulobacter* group. Bacteriol Rev **28**:231–295.

6. **Eaton DS**, **Crosson S**, **Fiebig A**. 2016. Proper Control of Caulobacter crescentus Cell Surface Adhesion Requires the General Protein Chaperone DnaK. J Bacteriol **198**:2631–2642.

7. **Aakre CD**, **Phung TN**, **Huang D**, **Laub MT**. 2013. A Bacterial Toxin Inhibits DNA Replication Elongation through a Direct Interaction with the β Sliding Clamp. Mol Cell **52**:617–628.
